# Neuroprotective Effects of Dexamethasone in a Neuromelanin-Driven Parkinson’s Disease Model

**DOI:** 10.1007/s11481-024-10164-4

**Published:** 2024-12-14

**Authors:** M. Garcia-Gomara, A. Juan-Palencia, M. Alfaro, M. Cuadrado-Tejedor, A. Garcia-Osta

**Affiliations:** 1 Gene Therapy for CNS Disorders Program, Center for Applied Medical Research (CIMA), University of Navarra, Pamplona, Spain; 2 IdiSNA (Navarra Institute for Health Research), Pamplona, Spain; 3 Department of Pathology, Anatomy and Physiology, School of Medicine, University of Navarra, Pamplona, Spain; 4 Immunology and Immunotherapy Program, Center for Applied Medical Research (CIMA), University of Navarra, Instituto de Investigación Sanitaria de Navarra (IdiSNA), Pamplona, Spain

**Keywords:** Parkinson’s disease, Neuroinflammation, Neuromelanin, Corticoids, Dexamethasone

## Abstract

Parkinson’s disease (PD) is characterized by the progressive loss of dopaminergic neurons in the substantia nigra that primarily affects movement control. Neuroinflammation plays a pivotal role in driving the disease’s progression. The persistent inflammatory state in the brain exacerbates neuronal damage, creating a cycle that perpetuates the neurodegenerative process. Glucocorticoids, such as dexamethasone, have potent anti-inflammatory properties and have been studied for their neuroprotective potential in different neurodegenerative diseases. However, their specific impact on PD remains unclear. This study aimed to evaluate the impact of dexamethasone on a neuromelanin (NM)-driven model of PD. We demonstrated that dexamethasone administration significantly improved motor function and preserved dopaminergic neuron compared to untreated controls in our study. These neuroprotective effects were mediated, at least in part, by suppressing reactive microglia and reducing the infiltration of peripheral immune cells into the brain. Our findings underscore the potential therapeutic benefits of dexamethasone in mitigating neuroinflammation and maintaining neuronal integrity in a NM-driven model of PD. These results advocate for further investigation into glucocorticoid-based therapies as adjunctive treatments for PD, particularly in scenarios where neuroinflammation contributes prominently to disease progression.

**Supplementary Information:**

The online version contains supplementary material available at 10.1007/s11481-024-10164-4.

## Introduction

Parkinson’s disease (PD) is characterized by a significant loss of pigmented neurons in the substantia nigra, leading to motor dysfunctions such as bradykinesia, stooped posture, and gait disturbances (Dickson 2018). This neuronal loss is accompanied by a pronounced microglial response, which may significantly contribute to the disease’s progression and could be a potential target for new therapeutic strategies (Block and Hong 2007; Imamura et al. 2003; Lavisse et al. 2021).

Microglia are central to neuroinflammatory responses in PD, releasing a variety of pro- and anti-inflammatory cytokines, antioxidants, and neurotrophic factors. The substantia nigra, along with the hippocampus, olfactory telencephalon, and basal ganglia, has the highest density of microglia in the adult mouse brain (Lawson et al. 1990). Recent studies have shown that midbrain microglia exhibit heightened vigilance and responsiveness to potential threats or environmental changes compared to microglia in other brain regions (Abellanas et al. 2019). Furthermore, microglia possess unique transcriptional profiles that vary by brain region and age differently across these regions (Grabert et al. 2016).

Two distinct populations of microglial cells have been identified in the midbrain: one expressing TLR4 and the other expressing MHC-II (Abellanas et al. 2019). MHC-II + microglia act as antigen-presenting cells (APCs) in the midbrain (Abellanas et al. 2019), a function seemingly specific to this brain region. As neuronal degeneration progresses in the substantia nigra of PD brains, there is a simultaneous increase in the population of microglia expressing MHC class II, both in human and animal models (Imamura et al. 2003; Martin et al. 2016). These activated microglia, producing TNF-alpha and interleukin-6, are known for their neuroprotective function, indicating that MHC class II-positive microglia are reliable indicators of neuropathological alterations and are directly linked to damaged neurons and neurites (Imamura et al. 2003). In fact, the genetic removal of MHC II provided neuroprotection to the cell bodies of dopaminergic neurons in the Substantia nigra pars compacta (SNpc) against MPTP toxicity (Martin et al. 2016).

One factor contributing to microglial activation in PD is NM, a complex molecule composed of melanin, peptides, and lipids, released by dying dopaminergic neurons. Being insoluble, NM remains in the extracellular space for a long time and is surrounded by or contained within activated microglia (Beach et al. 2007; Carballo-Carbajal et al. 2019). Microglial cells are primarily responsible for recognizing, engulfing, and clearing extracellular NM (Beach et al. 2007; Wilms et al. 2003). When added to neuron-glia cultures, human NM is phagocytosed and degraded by microglia, releasing inflammatory factors and reactive oxygen species, which cause neuronal death. Thus, NM may contribute to the vicious cycle between dying neurons and reactive microgliosis that drives PD progression (Wilms et al. 2003; Zecca et al. 2003).

The role of microglial activation in PD is complex and multifaceted, involving both neuroprotective and neurodegenerative processes. Understanding better in vitro these mechanisms may lead to novel therapeutic approaches targeting microglial function and inflammation in PD. Glucocorticoids (GCs), through glucocorticoid receptors (GRs), have strong anti-inflammatory and immunosuppressive effects. Transgenic mice deficient in GRs exhibited increased vulnerability of dopaminergic neurons to the toxin 1-methyl-4-phenyl-1,2,3,6-tetrahydropyridine (MPTP) (Morale et al. 2004).

In the brain, Dexamethasone inhibits microglial activation by suppressing MHC class II expression and decreasing COX-2 and iNOS production (Kiefer and Kreutzberg 1991). It also prevents microglial ramification and proliferation in vitro and induces lipocortin expression in microglia, which inhibits activation and provides neuroprotection (Minghetti et al. 1999). Therefore, dexamethasone is a potent agent that can modify the inflammatory aspect of neurodegeneration. In the MPTP-induced degeneration model, some studies have reported a beneficial effect of dexamethasone (Kurkowska-Jastrzȩbska et al. 2004), while others did not observe the same outcome (Aubin et al. 1998). The purpose of this study was to investigate the influence of Dexamethasone on the degenerative process in PD using the NM-accumulating mouse model with the hypothesis that inhibition of inflammation might exert a neuroprotective effect.

## Materials and Methods

### Animals and Vector Production

Six weeks-old female C57BL/6 mice were purchased from Envigo (Barcelona, Spain). Animals were housed 6 per cage with free access to food and water, and maintained in a temperature-controlled environment on a 12 h light-dark cycle. All procedures were carried out in accordance with the current European and Spanish regulations and was approved by the Ethical Commitee of the University of Navarra (protocol nº 110 − 21).

Recombinant adeno associated virus (AAV) serotype 9 expressing the human tyrosinase cDNA driven by the CMV promoter (AAV-hTyr) were produced at the Viral Vector Production Unit of the Autonomous University of Barcelona (UPV-UAB, Spain).

### Stereotaxic Surgery for Viral Administration

Animals were anesthetized with an intraperitoneal dose of 80/10 mg/kg of ketamine/xylazine and treated with the analgesic buprenorphine (Buprex) at a dose of 0.1 mg/kg. Animals were bilaterally injected with 1 µl of AAV-hTyr (8.94 × 10^12 genomic copies/ml) or same amount of empty vector at a rate of 0.2 µl/min into the SNpc using a 10 µl Hamilton Neuros syringe (model 1701 RN; Hamilton, Reno, NV) and a pump (Stoelting Co, Wood Lane, IL). The coordinates of the SNpc were calculated using the atlas of Paxinos and Watson: antero-posterior − 3.52 mm; half-side ± 1.25 mm; dorso-ventral − 4.0 mm (using bregma point as reference). Before the administration of the AAV9, the needle was left for 2 min at the injection site. Following the injection, the needle was left in place for 10 min, to avoid vector leakage, before withdrawing slowly the syringe. After surgery, animals were kept under constant monitoring with *ad libitum* access to food and water.

### Dexamethasone Treatment

Dexamethasone (Acofarma, Barcelona) was dissolved in ethanol 50% and diluted 1:10 in NaCl 0.9%. It was given intraperitoneally (ip) at a dose of 1 mg/Kg/day.

Animals were divided into 6 groups (*n* = 7–9 per group): a control group (G1) with AAV-empty injection and vehicle (50% Ethanol in 0.9% NaCl), and groups with AAV-hTyr injection treated with vehicle (G2) or Dexamethasone (1 mg/kg/day) (G3) daily from 7 days post-surgery for 15 days and euthanized at 3 weeks. To evaluate prolonged effects, groups G4 (AAV-empty, control), G5 (AAV-hTyr -vehicle) and G6 (AAV-hTyr-Dexamethasone) were treated similarly for 15 days, then on alternate days for another 15 days, and euthanized at 5 weeks post-surgery. The animals were weighed before injections to monitor weight loss. Dexamethasone was administered between 8 a.m. and 9 a.m. to align with circadian rhythms.

### Motor Behavior Assessment

All behavioral studies used in this study were carried out during light time. For the rotarod test, animals were placed on top of a revolving bar with a constant acceleration from 4 rpm to 40 rpm in 5 min. To acclimate the mice to the rotating bar, they were exposed to it for 30 s on the first day and for 1 min on the second day. On the third day (test day), the mice were placed on the rod for two consecutive trials, with a 30-minute rest between them. The results were expressed as the average latency to fall across the two trials.

The catalepsy test was used to assess muscular rigidity in mice. Animals were placed with their forepaws on a parallel bar that was 4 cm above the ground. The average time it took to move their forepaws and adjust their posture was recorded across three trials, with a one-minute rest period between each trial.

### Neutral Red Staining

Neutral Red staining enables the observation of viable neurons that harbor NM. This dye specifically colors nuclei and lysosomes red; however, it is only taken up by living cells, excluding those that are dead or in the process of dying. Thus, we can distinguish neurons that possess NM but have not yet begun the process of cell death.

Brain sections were first placed on the slides and dried for 48 h at room temperature. Afterwards, they were incubated in Neutral red solution (0.42%) for 1 min. Then, they were incubated for 1 min in ethanol 70%, washed in distilled water, incubated in 1% acetic acid and washed again in distilled water. Finally, brain slices were dehydrated in xylene and mounted using Immu-Mount™ (Thermo Fisher Scientific). Images were analyzed with ImageJ.

### Intracellular NM Quantification

Intracellular NM levels were quantified in AAV9-hTyr injected mice at five weeks post-AAV injection in 40-µm thick stained sections. In these sections, SNpc dopaminergic neurons were identified by the visualization of unstained NM brown pigment. Midbrain sections were scanned using Aperio ScanScope software (Leica biosystems). All NM-positive neurons in a representative SNpc middle section exhibiting high numbers of NM-containing neurons were analyzed by means of optical densitometry using ImageJ software to quantify the intracellular density of NM pigment.

### Immunohistochemistry

Animals were anesthetized under ketamine/xylazine (80/10 mg/kg) and perfused transcardially with saline solution (0.9% NaCl) during 5 min at a rate of 9.5 ml/min, followed by 4% paraformaldehyde (PFA; Panreac, Barcelona, Spain) in phosphate buffer during 15 min at the same rate. Mice brains were removed and immersion-fixed in PFA during 24 h and store in 30% sucrose/PBS at 4 °C. Coronal microtome Sect. (40 μm thick) were collected and stored in a cryoprotective solution (30% Ethylene Glycol pure, 30% glycerol, 20% PBS1X, 20% dH2O) at -20 °C until use. Brain sections were washed three times with PBS and endogenous peroxidase was inactivated by incubation for 15 min in 0.3% H2O2 (Appac) in methanol (Panreac). After washing with PBS, brain tissue was incubated with the blocking solution (PBS containing 0.5% Triton X-100 and 5% normal goat serum) for 1 h at room temperature. Sections were then incubated for 24 h at 4 °C with one of the following antibodies diluted in blocking solution: rabbit anti-tyrosine hydroxylase (TH; 1:1000; Merck Millipore #Ab152), rabbit anti-Iba1 (Iba1; 1:1000; Wako #019-19741), rat anti-CD3 (CD3;1:200; Invitrogen #14003282), rat anti-CD68 (CD68; 1:1000; Abcam #Ab53444), Anti glial fibrillary acidic protein (GFAP; 1:500; Sigma #G9269). After washing with PBS, the brain sections were incubated with the secondary polyclonal goat anti-mouse biotinylated antibody (1:500, Dako #P0447), goat anti-rat biotinylated antibody (1:500, Sigma #B7139) or goat anti-rabbit biotinylated antibody (1:500, Dako #P0260) for 1 h and then with horseradish peroxidase (HRP)-labeled Vectastain Elite ABC kit (Vector Laboratories). The peroxidase reaction was visualized with 0.05% 3 − 3′-diaminobenzidine tetrahydrochloride (DAB, Vector Laboratories) in PBS or Vector VIP substrate kit (Vector Laboratories) for color development. Brain sections were mounted on gelatin-coated slides, air-dried, dehydrated in graded ethanol solutions, cleared in xylene and cover-slipped with DPX mounting medium (BDH Chemicals Ltd.). Images were analyzed with ImageJ software.

### Immunofluorescence

For immunofluorescence staining brain sections were blocked for 1 h with normal goat serum (NGS) and incubated 24 h at 4 °C with the following antibodies: rabbit anti-Iba1 (Iba1; 1:1000; Wako #019-19741), rat anti-CD68 (CD68; 1:1000; Abcam #Ab53444). After washing with PBS, the brain sections were incubated with the secondary antibodies diluted in blocking solution: Alexa Fluor 633 goat anti-rat (1:500; Invitrogen #A-21094), Alexa Fluor 568 goat anti-rabbit (1:500; Invitrogen #A11011). Sections were washed again with PBS and finally nuclei were stained with 4’,6-diamidino-2-phenylindole (DAPI, Sigma-Aldrich, 1:10.000 #D8417) during 15 min. The coverslips were placed on microscope slides on an 8-µL drop of mounting medium (DABCO; Sigma) and dried for 30 min at 37 °C.

### Stereological Quantification

The number of TH positive neurons present in the SNpc was determined by unbiased stereology. For the stereological quantification a Bx61 microscope (Olympus, Hicksville, NY) equipped with a camera DP71 (Olympus,), a stage connected to a xyz stepper (H101BX, PRIOR) and the Stereo Investigator software (version 2021.1.1; MBF Bioscience, Williston, VT) was used. Stereological counting was performed on 7 coronal SNpc Sect. (40 μm thick) taken at uniform intervals (120 μm) that covered the entire rostrocaudal extent of the nucleus between − 2.92 and − 3.64 mm relative to bregma (Paxinos & Franklin 2001). The optical dissector height was set at 11 μm to count 120–150 cells per animal using a sampling frame of 4900 µm2 and sampling steps of 140 μm × 140 μm (dx, dy). Unbiased counting was performed blindly and the total number of TH + neurons (N) was calculated.

Inmmunostained TH striatum sections were acquired on an Aperio CS2 Digital Pathology Slide Scanner (Leica) at a 20 × magnification. Optical density values were obtained using ImageJ softwere (National Institutes of Health, MD). A random region of the cortex was used as blank, and its value was subtracted to the average intensity of both hemispheres.

For Iba1, C68 + and CD3 + quantification, SN sections were acquired on an Aperio CS2 Digital Pathology Slide Scanner (Leica) at a 20× magnification. Values were obtained using Aperio ImageScope softwere (Leica biosystems Imaging, CA).

### Blood Preparation for Flow Cytometry

Blood samples were obtained using capillary tubes treated with heparin. Each tube was filled with approximately 60 µl of whole blood from individual mice. The blood samples were incubated for 15 min with the following antibodies: PE/Cyanine7 anti-mouse CD4 antibody (Clone RM 4–5, Biolegend #100528) for CD4 detection, FITC Hamster Anti-Mouse CD3e antibody (BD Pharmingen #553061) for CD3 detection and PerCP/Cyanine5.5 anti-mouse CD8a antibody (Clone 53 − 6.7, Biolegend #100733) for CD8 detection. The samples were washed with washing buffer and centrifuged for 5 min at 2200 rpm. Subsequently, the supernatant was discarded, and the samples were incubated with 700 µl of lysis buffer (BD FACS Lysing Solution #349202) for 30 min. Then they were washed with 500 µl of washing buffer and centrifuged again for 5 min at 2200 rpm. After discarding the supernatant, samples were transferred to cytometry tubes and 150 µl of counting beads (CountBright Plus Absolut Countin Beads, ThermoFisher #C36995) were added. Analyzed was done using a FACS Canto II flow cytometer (Becton Dickinson), and the data were processed with FlowJo software (TreeStar).

### Statistical Analysis

The results were processed for statistical analysis using GraphPad PRISM, version 8.02. Results are presented as mean ± standard error of the mean (SEM). Normal distribution of data was checked by the Shapiro–Wilk test. Unpaired two-tailed Student’s t-test was used to compare two groups and one-way ANOVA followed by Tukey post hoc test to compare more than two groups. Statistical significance was set at *p ≤ 0.05, **p ≤ 0.01, ***p ≤ 0.001****p ≤ 0.0001.

## Results

### Accumulation of NM in Nigrostriatal Dopaminergic Neurons Induces a Parkinson’s Disease Phenotype in Mice

**Fig. 1:**
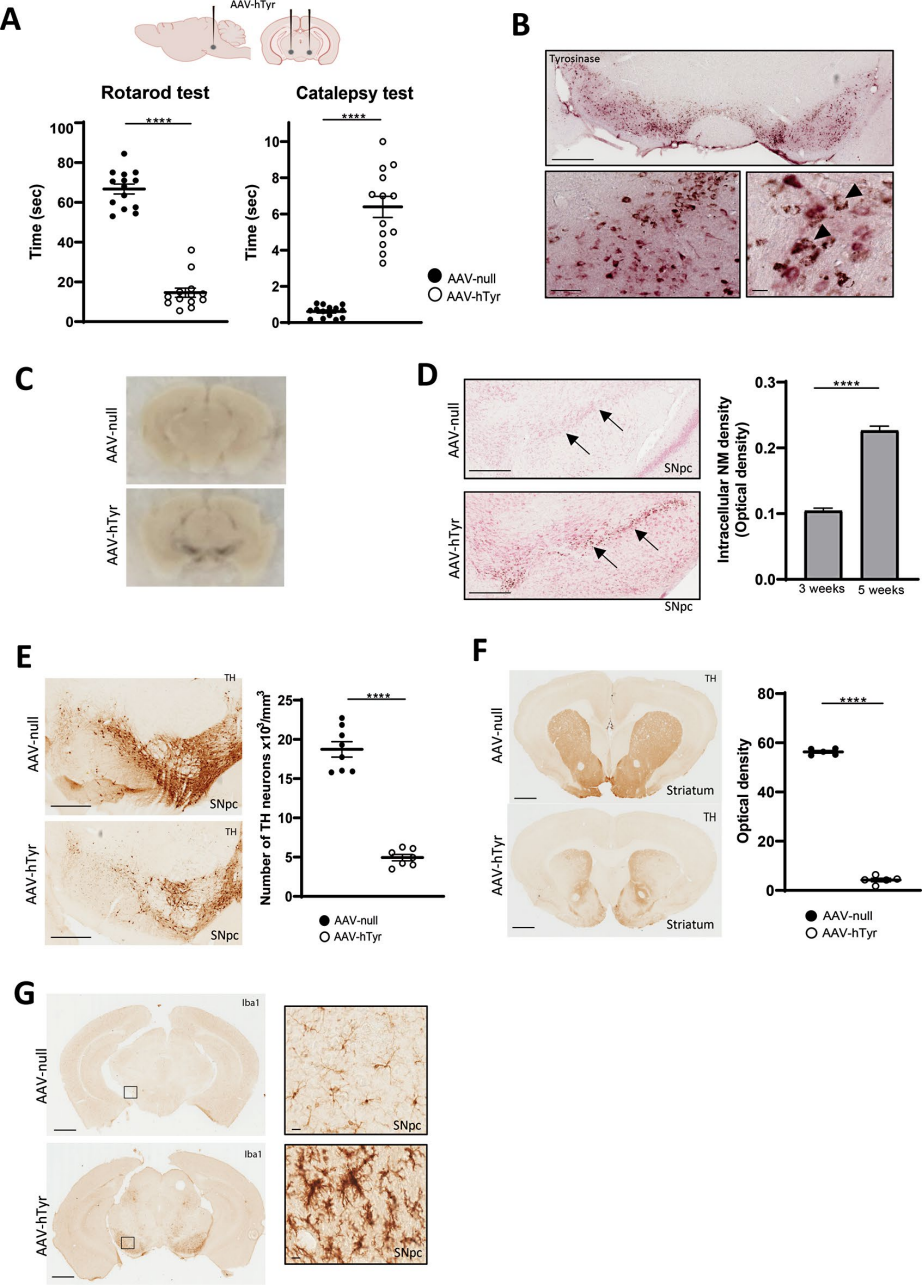
AAV9-mediated hTyr overexpression in the substantia nigra (SN) leads to motor deficits, DA neuronal loss and an inflammatory response in C57BL/6J mice three weeks after injection. (**A**) Motor function was evaluated 3 weeks after the AAV9-null or AAV9-hTyr injection in the SN of C57BL/6J mice (*n* = 14 − 13 per group). Motor coordination was assessed by measuring the latency to fall in the accelerating rotarod test (Rotarod test). Muscular rigidity was evaluated by measuring the time required for the mice to withdraw their forepaws from a bar (Catalepsy test). (**B**) Representative images of a SNpc section from an AAV-hTyr-injected mouse (3 weeks after AAV injection) immunostained for hTyr. NM was visualized macroscopically (**C**) and microscopically (**D**) in the SNpc of an AAV-hTyr-injected mouse 3 weeks after AAV9-hTyr injection. Quantification of intracellular NM optical density in DA neurons of mice at 3 and 5 weeks after AAV9-hTyr injection (*n* = 130–135 neurons). (**E**) Representative photomicrographs showing TH + dopaminergic neurons and quantification in the midbrain of C57BL/6J mice injected with AAV9-null and AAV9-hTyr (*n* = 6–8 per group). (**F**) Representative photomicrographs showing TH + fibers in the striatum and quantification measured by optical densitometry of C57BL/6J mice injected with AAV9-null and AAV9-hTyr (*n* = 6–8 per group). (**G**) Representatives images of Iba 1 immunostaining in the midbrain of C57BL/6J mice injected with AAV9-null or AAV9-hTyr. Magnification bar (**B**), (**D**), (**E**), (**F**) (**G**) 1 mm, and 200 μm in sets with high magnification. Data are presented as mean ± standard error of the mean (SEM). Unpaired two tailed Student´s t-test was used. ****p ≤ 0.0001

Given the significant recent advances in modelling PD through the use of rodents producing NM (Carballo-Carbajal et al. 2019), we employed an AAV9-hTyr vector, which was directly injected in the SNpc of C57BL6/6J mice to mimic a PD phenotype. The AAV-hTyr or empty AAV-null (control) vector was infused into both sides of the SNpc and 5-weeks later their motor behavior was evaluated in the rotarod and catalepsy tests. As expected, AAV-hTyr injected mice exhibited a shorter time latency to fall from the accelerating rotarod compared to their control (AAV-null) (*P* < 0.0001, Fig. 1A). Similarly, the time to start movement in the catalepsy test increased significantly in the AAV-hTyr injected mice relative to those that received AAV-null (*P* < 0.0001, Fig. 1A), indicating that AAV-hTyr injected mice developed a clear PD-like phenotype. Prior to analyzing the nigrostriatal pathway in these animals, hTyr expression in the substantia nigra pars compacta (SNpc) was confirmed by immunohistochemistry (Fig. 1B) and NM production was evidenced both macroscopically (Fig. 1C) and microscopically using neutral red staining (Fig. 1D). Next, we compared the amount of intraneuronal NM pigment stained with neutral red in the brains of mice at 3- and 5- weeks post AAV-Tyr injection. Figure 1D shows that mice injected with hTyr for 5 weeks had significantly more intracellular NM accumulation in the catecholaminergic neurons of the SNpc than those injected for 3 weeks.

Next, to determine whether overexpression of hTyr affected nigrostriatal dopaminergic neurons, an unbiased stereology method was used to estimate the number of tyrosine hydroxylase-positive neurons (TH+) in the SNpc. Representative photomicrographs of TH + neurons are shown in Fig. 1E. Stereological cell counts revealed a significant loss of TH-positive neurons of SNpc in AAV-hTyr-injected mice compared to AAV-null-injected group (*P* < 0.0001, Fig. 1E). A Nissl staining was also achieved to confirm that stereological cell counts of TH + neurons really represent a significant neuronal loss rather than a decrease in TH expression (Suppl Fig. 1).

Likewise, striatal tissue sections were also processed for immunohistochemistry to determine whether the loss of nigrostriatal neurons resulted in decreased TH + axons in their target region. Interestingly, a reduction of striatal DA TH-positive fibers measured by optical densitometry, was also patent in AAV-hTyr injected mice compared to controls (AAV-null) (Fig. 1F, *P* < 0.001), that correlates with the significant motor deficits that these animals displayed. Finally, considering that neuroinflammation and microglia activation are considered neuropathological hallmarks in PD, AAV-hTyr and AAV-null mice brains were immune-stained for Iba-1. As expected, the SNpc of hTyr injected animals showed an important microglial response, whereas the injection of the null vector produced minimal microglial activation (Fig. 1G). The increase of Iba-1 + cells in the SNpc of AAV-hTyr- injected mice suggests that the NM accumulation induces a robust inflammatory response. This finding is consistent with previous studies indicating that NM released from dying neurons can activate microglia, thereby promoting the degeneration of neighboring neurons (Zecca et al. 2003).

### Dexamethasone Ameliorates Behavioral Deficits Exhibited by NM-PD Mice

Given the significant neuroinflammation observed in our model and the consensus among several authors that inflammation may contribute to dopaminergic neuron degeneration in PD, and thus to its progression, we propose to investigate the potential benefits of an anti-inflammatory therapy. Thus, leveraging the NM PD mouse model described in Fig. 1, we selected dexamethasone, a potent glucocorticoid, as a potential candidate to reduce brain inflammation and protect dopamine-producing neurons from degeneration.

**Fig. 2:**
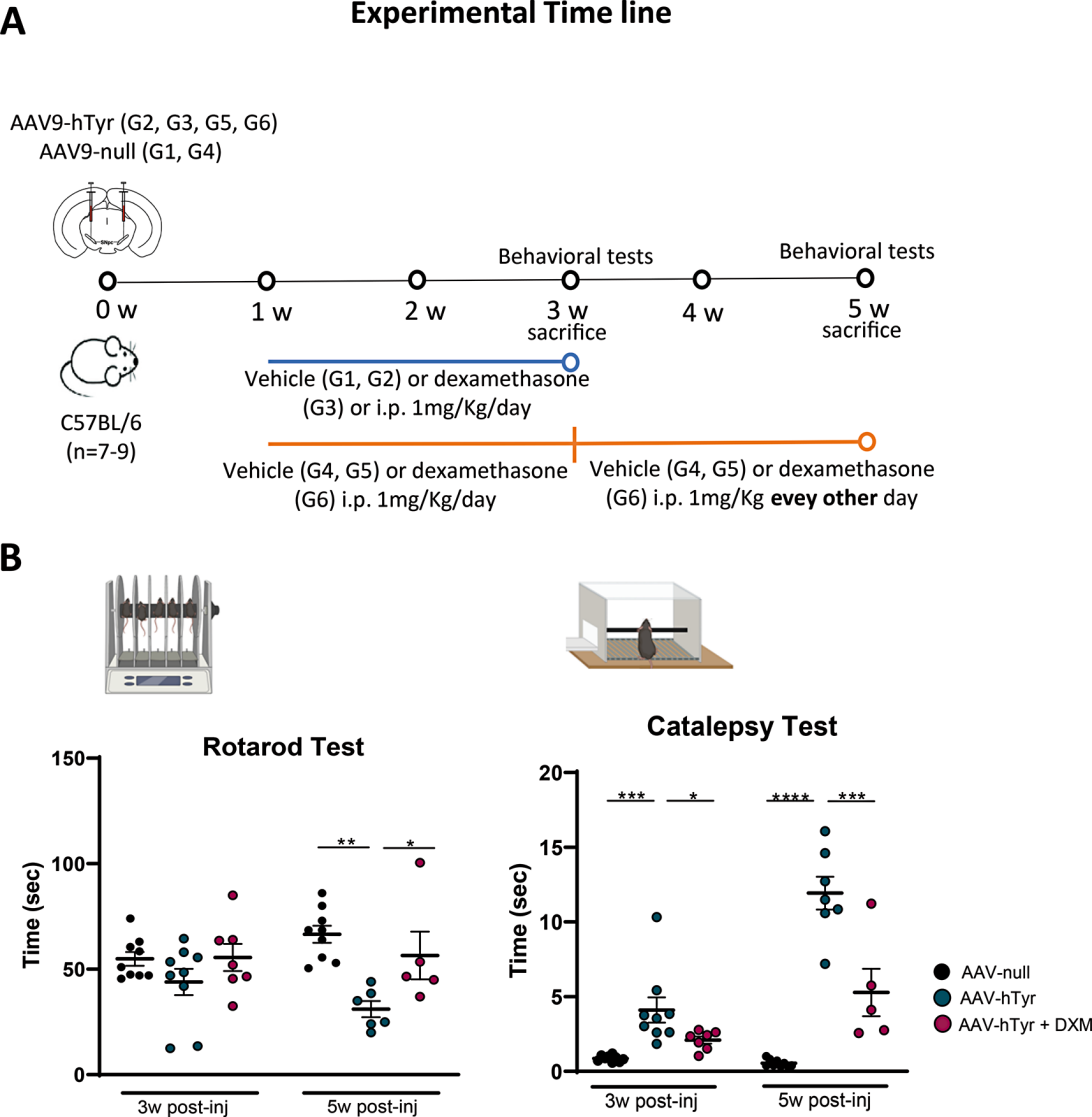
Dexamethasone (DXM) significantly ameliorates the behavioral deficits exhibited by NM-PD mice. (**A**) Timeline of AAV Administration, motor testing, and procedures in different experimental groups of C57BL/6J mice over 5 weeks. (**B**) Motor coordination was analyzed by measuring the latency to fall in accelerating rotarod test (Rotarod test) at 3- and 5- weeks post-injection. Muscular rigidity was evaluated by measuring the time required for the mice to withdraw their forepaws from a bar (Catalepsy test) at 3- and 5- weeks post-injection. (*n* = 5–9 per group). Data are presented as mean ± standard error of the mean (SEM). One-way ANOVA followed by Tukey post hoc test was used. *p ≤ 0.05, **p ≤ 0.01, better ***p ≤ 0.001****p ≤ 0.0001

Considering that NM levels are detectable at 3 weeks, with a significant increase observed at 5 weeks, where we have confirmed the establishment of a severe PD phenotype (Fig. 1) two schedules of dexamethasone or vehicle treatment (2- and 4-week treatment starting 1-week after AAV9-hTyr injection9), were implemented (Fig. 2A). Two groups of mice injected with an empty AAV (AAV-null) and scarified 3- or 5 weeks post injection were used as controls. AAV9-hTyr mice, whether treated with vehicle or dexamethasone, exhibited comparable body weights at baseline and experienced anticipated weight gain over the subsequent 3 weeks of treatment. By the end of this period, mice receiving the vehicle displayed slightly higher weights compared to those receiving dexamethasone, though no significant differences between both groups were detected (Suppl. Figure 2A). Two behavioral tests (rotarod and catalepsy) were employed to measure motor function of control and AVV9-hTyr mice after 2- or 4-weeks of dexamethasone or vehicle treatment. In the rotarod test, no significant differences were observed among the groups at 3 weeks post-injection. However, by 5 weeks post-injection, the vehicle-AAV9-hTyr mice showed a significantly shorter latency to fall from the accelerating rotarod compared to the control group (*P* < 0.01) and compared to dexamethasone-treated AAV9-hTyr animals (*P* < 0.05, Fig. 2B). Most notably, the time to descend or initiate movement in the catalepsy test significantly increased progressively over time in the AAV-hTyr vehicle-treated mice compared to controls (*P* < 0.001 at 3 weeks and *P* < 0.0001 at 5 weeks post-injection, Fig. 2B). Interestingly, the AAV-hTyr injected mice that received 2 or 3 weeks of dexamethasone treatment showed significant improvements compared to the AAV-hTyr vehicle group at both time points (*P* < 0.05 at 3 weeks and *P* < 0.001 at 5 weeks post-injection, Fig. 2B). These data indicate that dexamethasone can alleviate the behavioral deficits exhibited by the NM PD mouse model.

### Dexamethasone Prevents the loss of Dopaminergic Neurons in the SNpc of NM-PD mice

**Fig. 3:**
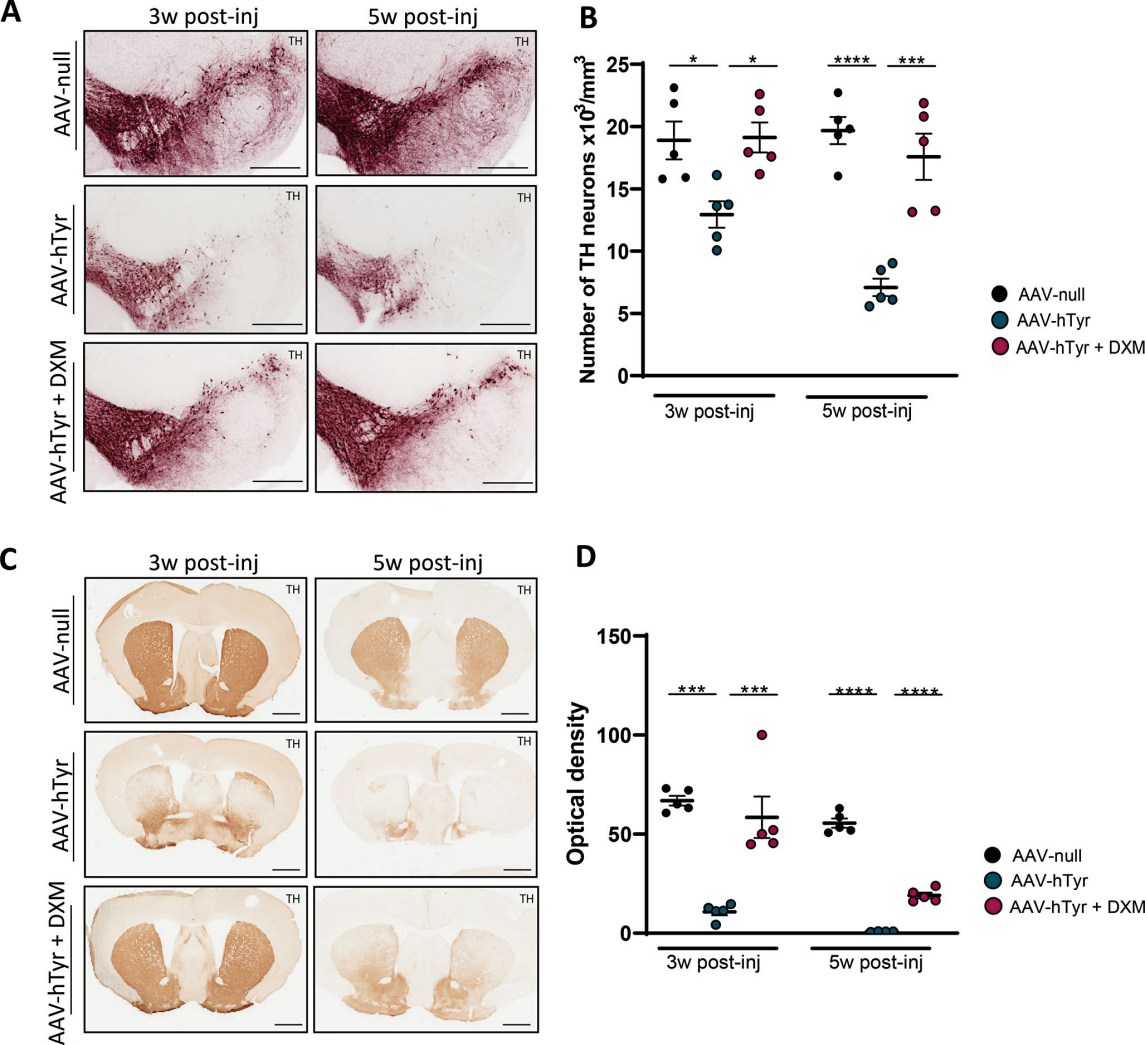
Dexamethasone prevents the loss of dopaminergic neurons in the SNpc of NM-PD mice. (**A**) Representative photomicrographs showing TH + dopaminergic neurons in the midbrain of C57BL/6J mice at 3 and 5 weeks following AAV9-null and AAV9-hTyr injections and treated with Dexamethasone (DXM) or vehicle. (**B**) Quantification of TH + neurons in the midbrain of C57BL/6J mice at 3 and 5 weeks following AAV9-null and AAV9-hTyr injections and treated with DXM or vehicle. (**C**) Representative photomicrographs showing TH-immunoreactivity in the striatum of C57BL/6J mice at 3 and 5 weeks following AAV9-null and AAV9-hTyr injections and treated with DXM or vehicle. (**D**) TH + fibers measured by optical densitometry in striatum of C57BL/6J mice at 3 and 5 weeks following AAV9-null and AAV9-hTyr and treated with DXM or vehicle (*n* = 5 per group). Magnification bar (**A**) 1 mm. Data are presented as mean ± standard error of the mean (SEM). One-way ANOVA followed by Tukey post hoc test was used.***p ≤ 0.001****p ≤ 0.0001

Dopaminergic cell death in the SNpc was measured in the experimental groups using unbiased stereology to estimate the number of TH + neurons in the SNpc (see Fig. 3A for representative photomicrographs). A one-way ANOVA analysis indicated that, at 3- and 5 weeks AAV9-hTyr injection, vehicle-treated mice exhibited a notable and progressive reduction in TH + cells within the SNpc compared to their respective control group (*P* < 0.05 and *P* < 0.0001 in AAV-null vs. AAV-Tyr at 3- and 5- weeks post-inj respectively). Remarkably, as shown in Fig. 3B, both 2- (*P* < 0.05) and 4-week (*P* < 0.001) dexamethasone treatments effectively and significantly prevented the decline of DA neurons caused by the overexpression of hTyr. To ensure that the neuroprotective effect was not due to dexamethasone reducing the AAV-mediated expression of hTyr, we performed a real-time PCR analysis. The results confirmed that the enzyme levels were similar in both the vehicle-treated and dexamethasone-treated groups (Suppl. Figure 2).

Next, striatal sections (see Fig. 3C for representative photomicrographs) were examined by immunohistochemistry, finding a significant reduction in striatal DA TH + fibers measured by optical densitometry in vehicle AAV-hTyr injected mice compared to control group at both time points (*P* < 0.001 and *P* < 0.0001 in AAV-null vs. AAV-Tyr at 3- and 5- weeks post-inj respectively Fig. 3D). Interestingly, 3-weeks after the injection of AAV-hTyr, dexamethasone is able to almost completely preserve DA fibers from neurodegeneration (*P* < 0.001). Significantly, at 5 weeks post-injection, the intensity of TH + immunoreactivity in the Tyr-dexamethasone group was notably higher compared to the Tyr-vehicle group. However, it is important to note that the disease has progressed despite the treatment, probably indicating that the anti-inflammatory effect had less impact on the terminals compared to the cell bodies (Fig. 3D). These findings, while highlighting the potential impact of anti-inflammatory treatment on modulating the degenerative cascade, underscore the complexity of neurodegenerative processes and the challenges in maintaining therapeutic effects with anti-inflammatory drugs over prolonged time periods.

### Dexamethasone Inhibits AAV9-hTyr-induced Microglia Activation in the Substantia Nigra pars Compacta

**Fig. 4:**
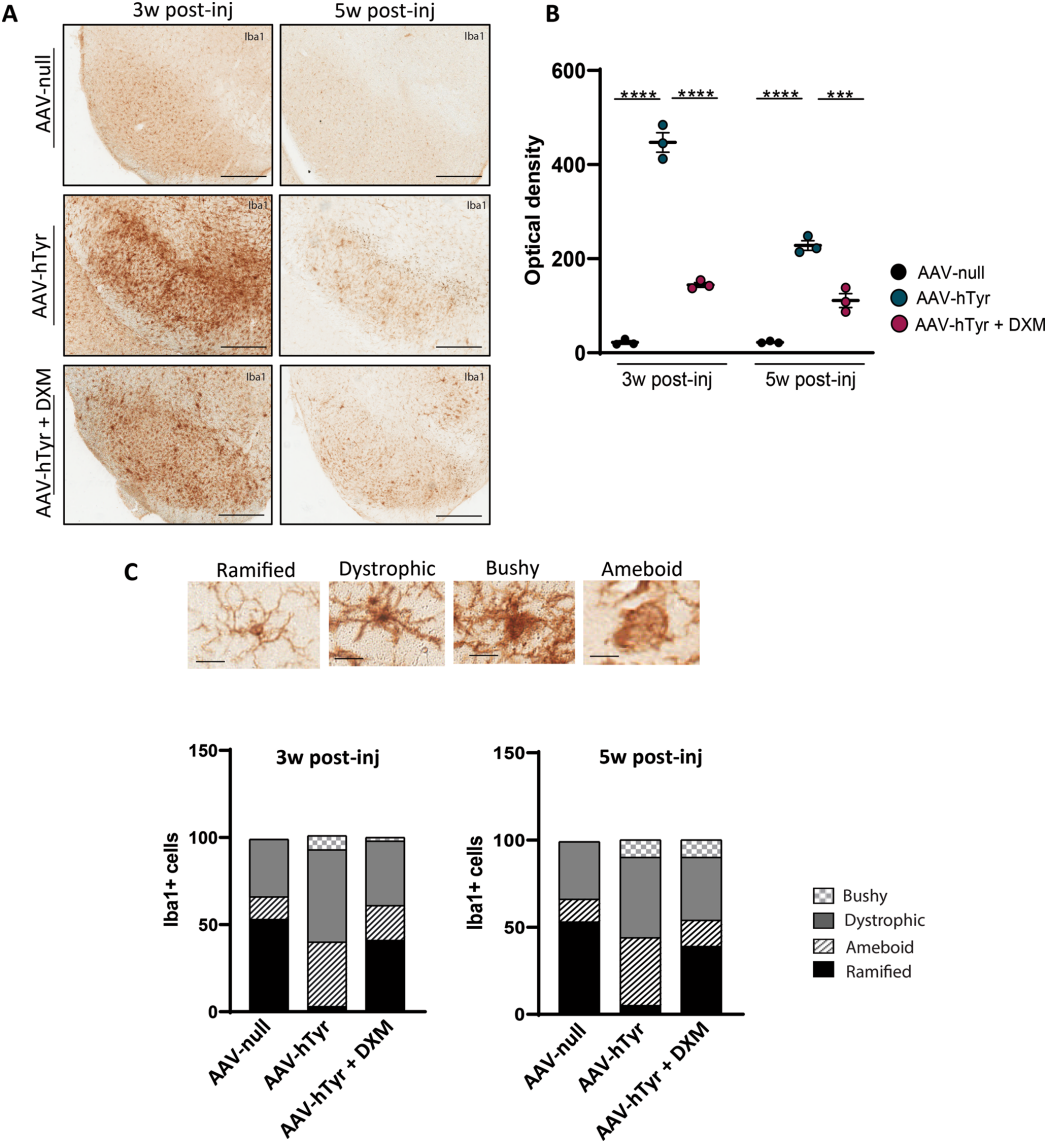
Dexamethasone inhibits AAV9-hTyr-induced microglia activation in the SNpc of C57BL/6J mice at 3- and 5- weeks post-injection. (**A**) Representative images of Iba1 immunostaining in the midbrain of C57BL/6J mice 3 and 5 weeks after AAV9-null or AAV9-hTyr injection and treated with Dexamethasone (DXM) or vehicle. (**B**) Iba1-immunoreactivity in the SN of C57BL/6J mice 3 and 5 weeks after the AAV9-null and AAV9-hTyr injection and treated with DXM or vehicle (**C**) Representative images of iba1 + cells with different phenotypes (ramified, dystrophic, ameboid and bushy) in C57BL/6J mice injected with AAV9-null, AAV9-hTyr and AAV9-hTyr treated with DXM at 3 weeks post-injection (upper panel). Ramified phenotype is characterized by long and thin ramifications with apparent cell integrity, dystrophic cells exhibit thicker and less clear ramifications, ameboid microglia are distinguished by the absence of ramifications and bushy cells posse a large nucleus and shorter ramifications (upper panel). Classification and quantification of Iba + cells in the midbrain of C57BL/6J mice 3 and 5 weeks after the AAV9-null and AAV9-hTyr injection and treated with DXM or vehicle (lower panel) (*n* = 5 per group). Magnification bar (**A**) 1 mm (**C**) 100 μm. Data are presented as mean ± standard error of the mean (SEM). One-way ANOVA followed by Tukey post hoc test was used. ***p ≤ 0.001****p ≤ 0.0001

Considering that dexamethasone reduces microglial activation associated to neuroinflammation (Hinkerohe et al. 2010; Hui et al. 2020a), microglia were next analyzed by immunohistochemistry using Iba-1 as the classical microglial marker in the experimental groups (Fig. 4A). As expected, at 3-weeks post injection, a marked increase in immunoreactivity for Iba-1 in the SNPc (Fig. 4B) was observed in the AAV9-hTyr-vehicle group compared to control group (AAV-null), indicative of the microglial response to damage and disease (Gao et al. 2014). The inflammatory response was significantly higher at 3 weeks post-viral injection compared to 5 weeks, likely corresponding to the peak of neurodegeneration, when a substantial amount of extracellular NM is released, triggering microglial reactivation. Interestingly, dexamethasone significantly mitigated Iba1 immunoreactivity at 3 and 5 weeks post-AAV9-hTyr injection (Fig. 4B).

We subsequently assessed the extent of Iba1-expressing microglial cells within the striatum; however, no significant differences were observed between groups at any time point (Suppl. Figure 3). Astroglial activation, characterized by GFAP expression, showed an increase in the number of immunoreactive cell bodies in the AAV-Tyr group at 3 weeks post-injection, which was attenuated with Dexamethasone treatment (Suppl Fig. 4). At 5 weeks post-injection, astrocyte activation was less pronounced, and no significant differences were detected between the treated and untreated AAV-Tyr groups.

Next, considering that distinctive alterations in microglial morphology, such as changes in cell shape, branching patterns, and soma size, can signify different stages of microglial activation and may correlate with disease progression and severity, we explored the morphology of these Iba1 + cells. A meticulous analysis of different forms of microglia was counted at the SNpc of the experimental groups based in a recent study (Basurco et al. 2023). Basurco et al., identified four distinct morphological types of microglia: ramified, hypertrophic, bushy, and ameboid. As displayed in Fig. 4C, the ramified phenotype is characterized by long and thin ramifications, in contrast, to dystrophic cells, which exhibit thicker and less clear ramifications. Ameboid microglia, are distinguished by the absence of ramifications. Conversely, bushy cells possess a large nucleus and shorter ramifications. Specifically, in a total of 100 cells counted per group, our results indicate that control animals predominantly exhibit ramified microglia, representing 53% of the phenotype, followed by 33% dystrophic, and 13% ameboid phenotypes (Fig. 4B). In contrast, vehicle-treated animals, at both 3 and 5 weeks post-hTyr injection, showed only 3–5% ramified cells, higher percentage of dystrophic (46–53%) and ameboid (37–39%) phenotype, and 8–10% bushy cells. Interestingly, Dexamethasone-treated animals display a microglial morphology pattern more similar to that of control (AAV-null) animals, with a greater number of ramified cells (39–41%) and less dystrophic (36–37%) and ameboid (15–20%) cells than the vehicle groups. It is noteworthy that bushy microglia are observed solely in hTyr-injected animals treated with vehicle. Overall, the results suggest that vehicle-treated animals with PD-like pathology display a significant shift towards dystrophic and ameboid phenotypes, indicating an activated and potentially neurodegenerative state. Dexamethasone, on the other hand, appears to mitigate this microglial activation, maintaining a microglial phenotype closer to that of healthy controls.

**Fig. 5:**
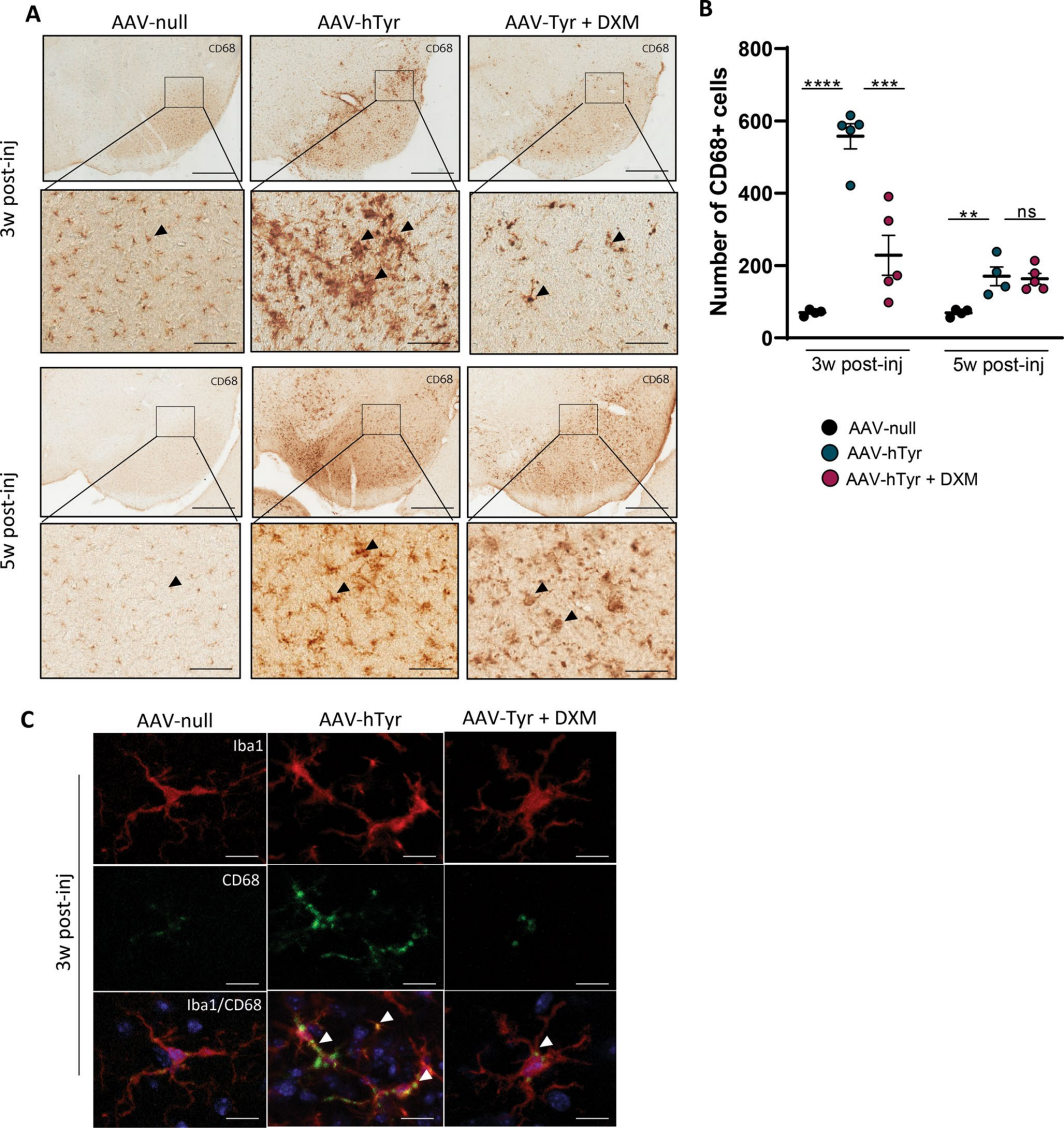
Dexamethasone inhibits AAV9-hTyr-induced activated phagocytic microglia in the SNpc of C57BL/6J mice at 3 weeks post-injection. (**A**) Representative photomicrographs showing CD68 + cells in the midbrain of C57BL/6J mice 3 and 5 weeks after the AAV9-null and AAV9-hTyr injection and treated with Dexamethasone (DXM) or vehicle. (**B**) Quantification of CD68 + cells in the midbrain of C57BL/6J mice 3 and 5 weeks after the AAV9-null and AAV9-hTyr injection and treated with DXM or vehicle. (**C**) Representative images of double labelling of Iba1 (red) and CD68 (green) of C57BL/6J mice 3 weeks after AAV9-null and AAV9-hTyr injection and treated with DXM or vehicle. Magnification bar (**A**) 1 mm in sets with low magnification and 200 μm in sets with high magnification. Data are presented as mean ± standard error of the mean (SEM). One-way ANOVA followed by Tukey post hoc test was used. **p ≤ 0.01 ***p ≤ 0.001****p ≤ 0.0001

To validate and confirm these findings, we conducted an additional analysis using CD68 to label activated phagocytic microglia (Walker and Lue 2015). Similar to Iba-1 expression, AAV9-hTyr injection induced a marked CD68 immunoreactivity in the substantia nigra that again was more pronounced at 3 weeks post-viral injection compared to 5 weeks (Fig. 5A). Interestingly, dexamethasone significantly mitigated CD68 immunoreactivity at 3 weeks post-AAV9-hTyr injection, but no significant differences were observed between the vehicle and dexamethasone groups at 5 weeks (Fig. 5B). Next, a double immunofluorescence staining of Iba1 and CD68 was performed at the earliest time point. The results showed that in Tyr-injected animals treated with the vehicle, a higher CD68 immunoreactivity was detected in the cell body of Iba1 + cells, indicating a phagocytic microglial phenotype. In contrast, the immunoreactivity for CD68 and the co-localization of both markers was scarce in dexamethasone-treated animals (Fig suppl 5 and Fig. 5C).

**Fig. 6:**
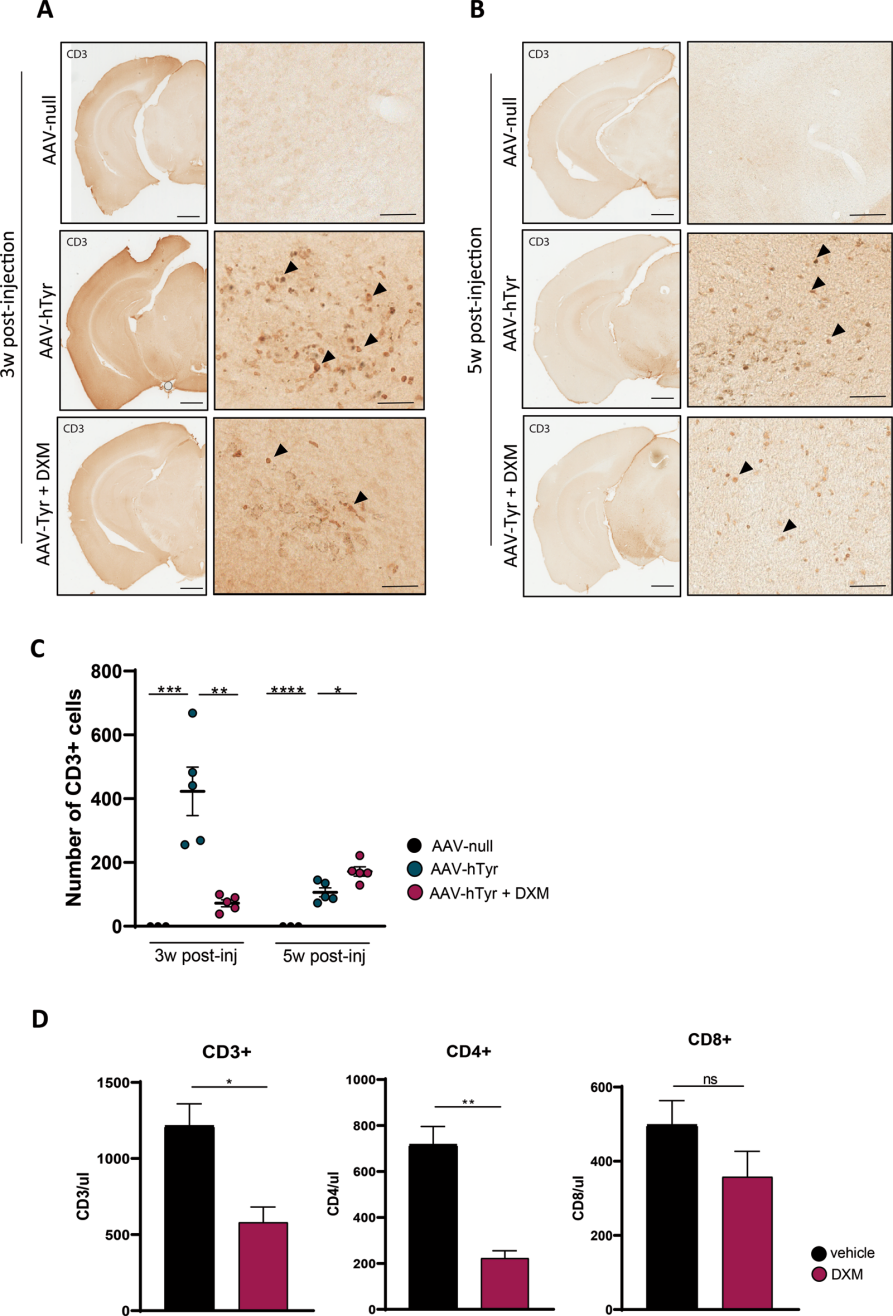
**Dexamethasone inhibits AAV9-hTyr-induced brain extravasation of lymphocytes in the SNpc.** Representative images of CD3 immunostaining in the midbrain of C57BL/6J mice 3 (**A**) and 5 (**B**) weeks after the AAV9-null and AAV9-hTyr injection and treated with Dexamethasone (DXM) or vehicle. (**C**) Quantification of CD3 + cells in the midbrain of C57BL/6J mice 3 and 5 weeks after the AAV9-null and AAV9-hTyr injection and treated with DXM or vehicle. (**D**) Quantification of CD3+, CD4 + and CD8 + cells in peripheral blood of C57BL/6J mice after treatment with DXM (1 mg/kg) or vehicle during 15 days (*n* = 4 per group). Data are presented as mean ± standard error of the mean (SEM). One-way ANOVA followed by Tukey post hoc test was used.*p ≤ 0.05, **p ≤ 0.01. Magnification bar 1 mm and 200 μm in sets with high magnification

Finally, considering that under pathological conditions, microglial cells may contribute to the recruitment of peripheral leukocytes (mostly T cells) into the brain (McGeer et al. 1993) (McGeer et al., reference), we investigated whether CD3 + T cells were also observed in the brains of hTyr-injected animals. Interestingly, significant brain extravasation of lymphocytes, evidenced by CD3 + immunostaining, was observed in the SNpc of hTyr-injected animals, mainly at 3 weeks (Fig. 6A) and much less evident at 5 weeks (Fig. 6B) post injection. In contrast, the SNpc of control animals and other brain regions of hTyr-injected mice were devoid of T cell infiltration (data not shown). Notably, Dexamethasone treatment resulted in a substantial decrease in CD3 + cells in the SNpc at 3 weeks post injection. Surprisingly, at 5 weeks post-injection, a significantly higher number of CD3 + cells were detected in the Dexamethasone group compared to the vehicle group (Fig. 6C). This suggests that while the neurodegenerative process persists, it progresses more slowly.

Given the immunosuppressive effects of glucocorticoids, the reduced CD3 + infiltration observed in the brain at 3 weeks post-injection (following 15 days of treatment) may result from treatment-induced depletion of peripheral CD3 + cells. To determine whether the dexamethasone dose we used impacted immune cells in the blood, we administered 1 mg/kg of dexamethasone to C57BL/6 mice for 15 consecutive days and assessed the abundance of peripheral T cells by flow cytometry. Notably, as shown in Fig. 6D, we observed a significant reduction in CD3 + and CD4 + cells in the blood of dexamethasone-treated C57BL/6J mice compared to the untreated group. These findings indicate that Dexamethasone attenuates microglial activation and slows the infiltration of peripheral immune cells, particularly CD3 + T cells, into the brain, thereby slowing the progression of the disease.

## Discussion

In the present study, we utilized a *murine* PD model based on the effects of hTyr-induced NM in the SNpc, which led to the development of motor symptoms and neurodegeneration in the nigrostriatal pathway. The accumulation of pigmentation via viral delivery of hTyr aligns with previous research on the SN in rats (Carballo-Carbajal et al. 2019; Gonzalez-Sepulveda et al. 2023). In this model, a significant neuroinflammatory response is associated with the release of extracellular NM from dying neurons. Here we observed motor dysfunction, neurodegeneration and neuroinflammation as early as three weeks post-AAV-hTyr injection, which progressively worsened by five weeks. Multiple studies have shown that persistent inflammatory responses, including glial cell activation and T cell infiltration, are prevalent in the brain of both human PD patients and animal models of the disease (Brochard et al. 2009; Hirsch et al. 2012; Wang et al. 2015). In fact, several authors have described that dopaminergic neurons are particularly sensitive to the toxic effects of pro-inflammatory mediators and subsequent oxidative, identifying neuroinflammation as one of the earliest and most significant characteristics of PD (Surmeier 2018). Consequently, several studies have proposed that inhibiting neuroinflammation could be a potential treatment for PD, although conclusive results have yet to be obtained. Given that this NM-mouse model effectively mimics PD features, making it a valuable tool for testing neuroprotective therapies targeting inflammatory processes, we aimed to use the synthetic glucocorticoid dexamethasone, a classic and potent anti-inflammatory drug, as a proof of concept to test this hypothesis.

Glucocorticoids are well known for their broad ranges of anti-inflammatory effects and have been widely used in clinical studies for brain inflammation. Certainly, dexamethasone treatment has been investigated in PD models, but the results have been inconsistent. It partially prevented neuronal loss caused by MPTP intoxication and reduced the inflammatory response, suggesting its anti-inflammatory properties are crucial to this protective effect (Kurkowska-Jastrzȩbska et al. 2004). Conversely, a subcutaneous single administration of dexamethasone, by suppressing microglial phagocytic elimination of glutamatergic synapses in the substantia nigra, aggravated motor deficits in the 6-OH-DA PD model as observed in rotarod and open field tests (Aono et al. 2017).

In this study utilizing the NM-accumulating PD model, we demonstrated that motor dysfunction, neurodegeneration, and neuroinflammation observed at three weeks post-injection were significantly attenuated in mice treated with dexamethasone for two weeks compared to those receiving vehicle treatment. This time point is likely coinciding with the peak of neuronal death and the highest release of NM, which triggers microglial activation in this model. The treatment mitigated the reactivation of microglial cells presented with both resting (ramified) and active/phagocytic morphologies (large amoeboid de-ramified and produce reactive species and pro-inflammatory mediators), which are associated with extracellular NM and neuronal death. Prolonged activation of the microglia contributes to the progression of neuronal damage (Simpson and Oliver 2020; Smith et al. 2012). Dexamethasone inhibits the production of COX-2 and phospholipase A2, reduces levels of several pro-inflammatory cytokines including IL-1β, IL-6, INFγ, and TNFα, and prevents the reactivation of microglial cells (Hui et al. 2020b; Newton et al. 1998). This mechanism helps to prevent the perpetuation and exacerbation of the neurodegenerative process. Since the inflammatory response appears to contribute to neuronal death in this model, the reduction of microglial activation by dexamethasone may be the primary mechanism through which it exerts its protective effect.

Additionally, dexamethasone administration prevented the activation of CD68-positive microglia and the infiltration of CD3-positive lymphocyte which are typically abundant near to melanized dopaminergic neurons in NM-producing animals, similar to observations in the brains of patients with PD (Brochard et al. 2009). Considering the detrimental role these cells play in disease progression, it is possible that the neuroprotective effect of dexamethasone is due to the induction of CD3 + cells depletion at the peripheral level. This is supported by our data (Fig. 6C) and previous findings showing that, in fact, dexamethasone induced a sustained drop in circulating T-lymphocytes (Miller and Schaefer 2007).

The inflammatory landscape in our model shifts at 5 weeks post-injection, when the substantia nigra has already lost the majority of dopaminergic neurons, both Iba1 and CD68 expression, and also CD3 + cells appear to be diminished in our model compared to 3 weeks post-injection. Thus, as inflammation becomes less significant in the neurodegenerative process, the effect of dexamethasone is not as pronounced at this time point. We can conclude that dexamethasone contains the inflammatory response when massive cell death is occurring, thereby slowing the neurodegenerative process. This is evidenced by the substantial preservation of TH + cell bodies in the substantia nigra observed five weeks after Tyr-injection in the dexamethasone-treated group compared to the untreated group. In contrast, protection of the dopaminergic nerve terminals in the striatum was only partial. This highlights the variability in dexamethasone’s effectiveness due to the differing roles of inflammation across brain regions. Thus, whereas in SN, where inflammation and degeneration are more pronounced, dexamethasone might offer greater protection to neuronal cells. In contrast, since neuroinflammation might not significantly contribute to the loss of striatal terminals, these areas might not benefit as much from dexamethasone’s protective effects. Similarly, in MHC II null mice, MPTP-induced toxicity spared neuronal cell bodies and reduced microgliosis but did not fully protect against the loss of dopaminergic nerve terminals in the striatum (Martin et al. 2016). Authors proposed that the reduced microgliosis in MHC II null mice may be due to the lack of CD4 + T-cell infiltration, that are the primary source of IFNγ, a key microglial activator. Consequently, reduced T-cell infiltration and IFNγ production might explain the observed decrease in microgliosis and associated neuroprotection (Martin et al. 2016). In any case, considering that SNpc neurons project to the striatum via the nigrostriatal pathway, the neuroprotective effects of Dexamethasone in the midbrain likely contribute to the preservation of dopaminergic nerve terminals in the striatum.

From a more translational perspective, we can say that prolonged use of non-steroidal anti-inflammatory drugs (NSAIDs), particularly ibuprofen, has been associated with a 21% reduction in the risk of PD, indicating significant therapeutic potential for NSAIDs in PD prevention (Gagne and Power 2010; Gao et al. 2011; Manthripragada et al. 2011). However, to date, trials conducted with anti-inflammatory drugs have not yielded positive results. The most recent trial with NLY0, a glucagon-like peptide-1 receptor agonist, believed to exert anti-inflammatory effects by reducing microglial activation, did not show any improvement in PD symptoms compared to placebo, except a possible motor benefits observed in the youngest patients (McGarry et al. 2024). These results indicate that inhibiting inflammatory signaling might not be sufficient to alter pathology and induce noticeable clinical effects, or might require a combination of interventions to achieve benefit.

To conclude, our study indicates that dexamethasone can delay the PD-like inflammatory response, thereby reducing neuronal death and the subsequent release of NM from degenerating neurons. Nonetheless, the disease continues to progress, albeit more slowly. However, more research is required to gain a better understanding of the detailed molecular mechanisms that contribute to the therapeutic effect as well as the stage of the disease to obtain benefit. Thus, while dexamethasone could serve as a therapeutic approach for treating PD, its efficacy might be significantly enhanced when used as part of a combined therapeutic strategy, potentially addressing multiple aspects of the disease and leading to better overall outcomes for patients.

## Electronic Supplementary Material

Below is the link to the electronic supplementary material.

Supplementary Material 1

## Data Availability

The data and materials supporting the conclusions of this study are available from the corresponding author on reasonable request.

## References

[CR1] Abellanas MA, Zamarbide M, Basurco L, Luquin E, Garcia-Granero M, Clavero P, Martin-Uriz S, Vilas P, Mengual A, Hervas-Stubbs E, Aymerich S, M.S (2019) Midbrain microglia mediate a specific immunosuppressive response under inflammatory conditions. J Neuroinflammation 16. 10.1186/S12974-019-1628-8 10.1186/s12974-019-1628-8 PMC6874825 31757220

[CR2] Aono H, Choudhury ME, Higaki H, Miyanishi K, Kigami Y, Fujita K, Akiyama JI, Takahashi H, Yano H, Kubo M, Nishikawa N, Nomoto M, Tanaka J (2017) Microglia may compensate for dopaminergic neuron loss in experimental parkinsonism through selective elimination of glutamatergic synapses from the subthalamic nucleus. Glia 65:1833–1847. 10.1002/GLIA.23199 28836295 10.1002/glia.23199

[CR3] Aubin N, Curet O, Deffois A, Carter C (1998) Aspirin and salicylate protect against MPTP-induced dopamine depletion in mice. J Neurochem 71:1635–1642. 10.1046/J.1471-4159.1998.71041635.X 9751197 10.1046/j.1471-4159.1998.71041635.x

[CR4] Basurco L, Abellanas MA, Ayerra L, Conde E, Vinueza-Gavilanes R, Luquin E, Vales A, Vilas A, Martin‐Uriz PS, Tamayo I, Alonso MM, Hernaez M, Gonzalez‐Aseguinolaza G, Clavero P, Mengual E, Arrasate M, Hervás‐Stubbs S, Aymerich MS (2023) Microglia and astrocyte activation is region-dependent in the α-synuclein mouse model of Parkinson’s disease. Glia 71 10.1002/GLIA.24295 10.1002/glia.24295 PMC10100513 36353934

[CR5] Beach TG, Sue LI, Walker DG, Lue LF, Connor DJ, Caviness JN, Sabbagh MN, Adler CH (2007) Marked microglial reaction in normal aging human substantia nigra: correlation with extraneuronal neuromelanin pigment deposits. Acta Neuropathol 114:419–424. 10.1007/S00401-007-0250-5/FIGURES/3 17639428 10.1007/s00401-007-0250-5

[CR6] Block ML, Hong JS (2007) Chronic microglial activation and progressive dopaminergic neurotoxicity. Biochem Soc Trans 35:1127–1132. 10.1042/BST0351127 17956294 10.1042/BST0351127

[CR7] Brochard V, Combadière B, Prigent A, Laouar Y, Perrin A, Beray-Berthat V, Bonduelle O, Alvarez-Fischer D, Callebert J, Launay JM, Duyckaerts C, Flavell RA, Hirsch EC, Hunot S (2009) Infiltration of CD4 + lymphocytes into the brain contributes to neurodegeneration in a mouse model of Parkinson disease. J Clin Invest 119:182–192. 10.1172/JCI36470 19104149 10.1172/JCI36470 PMC2613467

[CR8] Carballo-Carbajal I, Laguna A, Romero-Giménez J, Cuadros T, Bové J, Martinez-Vicente M, Parent A, Gonzalez-Sepulveda M, Peñuelas N, Torra A, Rodríguez-Galván B, Ballabio A, Hasegawa T, Bortolozzi A, Gelpi E, Vila M (2019) Brain tyrosinase overexpression implicates age-dependent neuromelanin production in Parkinson’s disease pathogenesis. Nat Commun 10. 10.1038/S41467-019-08858-Y 10.1038/s41467-019-08858-y PMC6405777 30846695

[CR9] Dickson DW (2018) Neuropathology of Parkinson disease. Parkinsonism Relat Disord 46(1):S30–S33. 10.1016/J.PARKRELDIS.2017.07.033 28780180 10.1016/j.parkreldis.2017.07.033 PMC5718208

[CR10] Gagne JJ, Power MC (2010) Anti-inflammatory drugs and risk of Parkinson disease: a meta-analysis. Neurology 74:995–1002. 10.1212/WNL.0B013E3181D5A4A3 20308684 10.1212/WNL.0b013e3181d5a4a3 PMC2848103

[CR11] Gao X, Chen H, Schwarzschild MA, Ascherio A (2011) Use of ibuprofen and risk of Parkinson disease. Neurology 76:863–869. 10.1212/WNL.0B013E31820F2D79 21368281 10.1212/WNL.0b013e31820f2d79 PMC3059148

[CR12] Gao Y, Ottaway N, Schriever SC, Legutko B, García-Cáceres C, de la Fuente E, Mergen C, Bour S, Thaler JP, Seeley RJ, Filosa J, Stern JE, Perez-Tilve D, Schwartz MW, Tschöp MH, Yi CX (2014) Hormones and Diet, but not body weight. Control Hypothalamic Microglial Activity Glia 62:17. 10.1002/GLIA.22580 24166765 10.1002/glia.22580 PMC4213950

[CR13] Gonzalez-Sepulveda M, Compte J, Cuadros T, Nicolau A, Guillard-Sirieix C, Peñuelas N, Lorente-Picon M, Parent A, Romero-Giménez J, Cladera-Sastre JM, Laguna A, Vila M (2023) In vivo reduction of age-dependent neuromelanin accumulation mitigates features of Parkinson’s disease. Brain 146:1040–1052. 10.1093/BRAIN/AWAC445 36717986 10.1093/brain/awac445 PMC9976971

[CR14] Grabert K, Michoel T, Karavolos MH, Clohisey S, Kenneth Baillie J, Stevens MP, Freeman TC, Summers KM, McColl BW (2016) Microglial brain region-dependent diversity and selective regional sensitivities to aging. Nat Neurosci 19:504–516. 10.1038/NN.4222 26780511 10.1038/nn.4222 PMC4768346

[CR15] Hinkerohe D, Smikalla D, Schoebel A, Haghikia A, Zoidl G, Haase CG, Schlegel U, Faustmann PM (2010) Dexamethasone prevents LPS-induced microglial activation and astroglial impairment in an experimental bacterial meningitis co-culture model. Brain Res 1329:45–54. 10.1016/J.BRAINRES.2010.03.012 20230803 10.1016/j.brainres.2010.03.012

[CR16] Hirsch EC, Vyas S, Hunot S (2012) Neuroinflammation in Parkinson’s disease. Parkinsonism Relat Disord. 10.1016/S1353-8020(11)70065-7. 18 Suppl 1 22166438 10.1016/S1353-8020(11)70065-7

[CR17] Hui B, Yao X, Zhang L, Zhou Q (2020a) Dexamethasone sodium phosphate attenuates lipopolysaccharide-induced neuroinflammation in microglia BV2 cells. Naunyn Schmiedebergs Arch Pharmacol 393:1761–1768. 10.1007/S00210-019-01775-3 31915845 10.1007/s00210-019-01775-3

[CR18] Hui B, Yao X, Zhang L, Zhou Q (2020b) Dexamethasone sodium phosphate attenuates lipopolysaccharide-induced neuroinflammation in microglia BV2 cells. Naunyn Schmiedebergs Arch Pharmacol 393:1761–1768. 10.1007/S00210-019-01775-3 31915845 10.1007/s00210-019-01775-3

[CR19] Imamura K, Hishikawa N, Sawada M, Nagatsu T, Yoshida M, Hashizume Y (2003) Distribution of major histocompatibility complex class II-positive microglia and cytokine profile of Parkinson’s disease brains. Acta Neuropathol 106:518–526. 10.1007/S00401-003-0766-2/FIGURES/6 14513261 10.1007/s00401-003-0766-2

[CR20] Kiefer R, Kreutzberg GW (1991) Effects of dexamethasone on microglial activation in vivo: selective downregulation of major histocompatibility complex class II expression in regenerating facial nucleus. J Neuroimmunol 34:99–108. 10.1016/0165-5728(91)90119-R 1918330 10.1016/0165-5728(91)90119-r

[CR21] Kurkowska-Jastrzȩbska I, Litwin T, Joniec I, Ciesielska A, Przybyłkowski A, Członkowski A, Członkowska A (2004) Dexamethasone protects against dopaminergic neurons damage in a mouse model of Parkinson’s disease. Int Immunopharmacol 4:1307–1318. 10.1016/j.intimp.2004.05.006 15313429 10.1016/j.intimp.2004.05.006

[CR22] Lavisse S, Goutal S, Wimberley C, Tonietto M, Bottlaender M, Gervais P, Kuhnast B, Peyronneau MA, Barret O, Lagarde J, Sarazin M, Hantraye P, Thiriez C, Remy P (2021) Increased microglial activation in patients with Parkinson disease using [18F]-DPA714 TSPO PET imaging. Parkinsonism Relat Disord 82:29–36. 10.1016/J.PARKRELDIS.2020.11.011 33242662 10.1016/j.parkreldis.2020.11.011

[CR23] Lawson LJ, Perry VH, Dri P, Gordon S (1990) Heterogeneity in the distribution and morphology of microglia in the normal adult mouse brain. Neuroscience 39:151–170. 10.1016/0306-4522(90)90229-W 2089275 10.1016/0306-4522(90)90229-w

[CR24] Manthripragada AD, Schernhammer ES, Qiu J, Friis S, Wermuth L, Olsen JH, Ritz B (2011) Non-steroidal anti-inflammatory drug use and the risk of Parkinson’s disease. Neuroepidemiology 36:155–161. 10.1159/000325653 21508649 10.1159/000325653 PMC3095838

[CR25] Martin HL, Santoro M, Mustafa S, Riedel G, Forrester JV, Teismann P (2016) Evidence for a role of adaptive immune response in the disease pathogenesis of the MPTP mouse model of Parkinson’s disease. Glia 64:386. 10.1002/GLIA.22935 26511587 10.1002/glia.22935 PMC4855685

[CR26] McGarry A, Rosanbalm S, Leinonen M, Olanow CW, To D, Bell A, Lee D, Chang J, Dubow J, Dhall R, Burdick D, Parashos S, Feuerstein J, Quinn J, Pahwa R, Afshari M, Ramirez-Zamora A, Chou K, Tarakad A, Luca C, Klos K, Bordelon Y, Hiliare S, Shprecher MH, Lee D, Dawson S, Roschke TM, Kieburtz V, K (2024) Safety, tolerability, and efficacy of NLY01 in early untreated Parkinson’s disease: a randomised, double-blind, placebo-controlled trial. Lancet Neurol 23:37–45. 10.1016/S1474-4422(23)00378-2 38101901 10.1016/S1474-4422(23)00378-2

[CR27] McGeer PL, Kawamata T, Walker DG, Akiyama H, Tooyama I, McGeer EG (1993) Microglia in degenerative neurological disease. Glia 7:84–92. 10.1002/GLIA.440070114 8423066 10.1002/glia.440070114

[CR28] Miller TA, Schaefer FW (2007) Changes in mouse circulating leukocyte numbers in C57BL/6 mice immunosuppressed with dexamethasone for Cryptosporidium parvum oocyst production. Vet Parasitol 149:147–157. 10.1016/J.VETPAR.2007.08.017 17904293 10.1016/j.vetpar.2007.08.017

[CR29] Minghetti L, Nicolini A, Polazzi E, Greco A, Perretti M, Parente L, Levi G (1999) Down-regulation of microglial cyclo-oxygenase-2 and inducible nitric oxide synthase expression by lipocortin 1. Br J Pharmacol 126:1307. 10.1038/SJ.BJP.0702423 10217523 10.1038/sj.bjp.0702423 PMC1565901

[CR30] Morale MC, Serra PA, Delogu MR, Migheli R, Rocchitta G, Tirolo C, Caniglia S, Testa N, L’Episcopo F, Gennuso F, Scoto GM, Barden N, Miele E, Desole MS, Marchetti B (2004) Glucocorticoid receptor deficiency increases vulnerability of the nigrostriatal dopaminergic system: critical role of glial nitric oxide. FASEB J 18:164–166. 10.1096/FJ.03-0501FJE 14630699 10.1096/fj.03-0501fje

[CR31] Newton R, Seybold J, Kuitert LME, Bergmann M, Barnes PJ (1998) Repression of cyclooxygenase-2 and prostaglandin E2 release by dexamethasone occurs by transcriptional and post-transcriptional mechanisms involving loss of polyadenylated mRNA. J Biol Chem 273:32312–32321. 10.1074/JBC.273.48.32312 9822711 10.1074/jbc.273.48.32312

[CR50] Paxinos G, Franklin KBJ (2001) The mouse brain in stereotaxic coordinates, 2nd edn. Academic Press,San Diego

[CR32] Simpson DSA, Oliver PL (2020) ROS Generation in Microglia: understanding oxidative stress and inflammation in neurodegenerative disease. Antioxid (Basel Switzerland) 9:1–27. 10.3390/ANTIOX9080743 10.3390/antiox9080743 PMC7463655 32823544

[CR33] Smith JA, Das A, Ray SK, Banik NL (2012) Role of pro-inflammatory cytokines released from microglia in neurodegenerative diseases. Brain Res Bull 87:10. 10.1016/J.BRAINRESBULL.2011.10.004 22024597 10.1016/j.brainresbull.2011.10.004 PMC9827422

[CR34] Surmeier DJ (2018) Determinants of dopaminergic neuron loss in Parkinson’s disease. FEBS J 285:3657–3668. 10.1111/FEBS.14607 30028088 10.1111/febs.14607 PMC6546423

[CR35] Walker DG, Lue LF (2015) Immune phenotypes of microglia in human neurodegenerative disease: challenges to detecting microglial polarization in human brains. Alzheimers Res Ther 7. 10.1186/S13195-015-0139-9 10.1186/s13195-015-0139-9 PMC4543480 26286145

[CR36] Wang Q, Liu Y, Zhou J (2015) Neuroinflammation in Parkinson’s disease and its potential as therapeutic target. Transl Neurodegener 4. 10.1186/S40035-015-0042-0 10.1186/s40035-015-0042-0 PMC4603346 26464797

[CR37] Wilms H, Rosenstiel P, Sievers J, Deuschl G, Zecca L, Lucius R (2003) Activation of microglia by human neuromelanin is NF-κB-dependent and involves p38 mitogen-activated protein kinase: implications for Parkinson’s disease. FASEB J 17:1–20. 10.1096/FJ.02-0314FJE 12631585 10.1096/fj.02-0314fje

[CR38] Zecca L, Zucca FA, Wilms H, Sulzer D (2003) Neuromelanin of the substantia nigra: a neuronal black hole with protective and toxic characteristics. Trends Neurosci 26:578–580. 10.1016/J.TINS.2003.08.009 14585596 10.1016/j.tins.2003.08.009

