# Supplementary material for: Neuroprotective Effects of Dexamethasone in a Neuromelanin-Driven Parkinson’s Disease Model
Source: J Neuroimmune Pharmacol. 2024 Dec 14;20(1):2. doi: 10.1007/s11481-024-10164-4 (PMC11645310; doi:10.1007/s11481-024-10164-4)
Supplement: Supplementary file 1 — Supplementary Material 1 [file 11481_2024_10164_MOESM1_ESM.pdf]

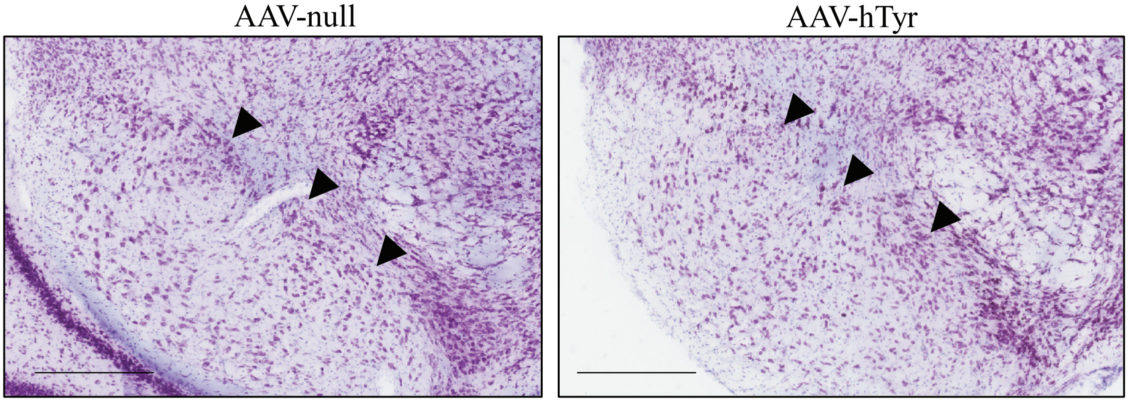

Suppl Figure 1. AAV9-mediated hTyr overexpression in the SN induces neuronal loss in C57BL6/J mice. Representative sections of the Nissl staining of brain sections containing the substantia nigra of AAV-null or AAV-hTyr C57BL6/J mice. Magnification bar (a) 1mm.

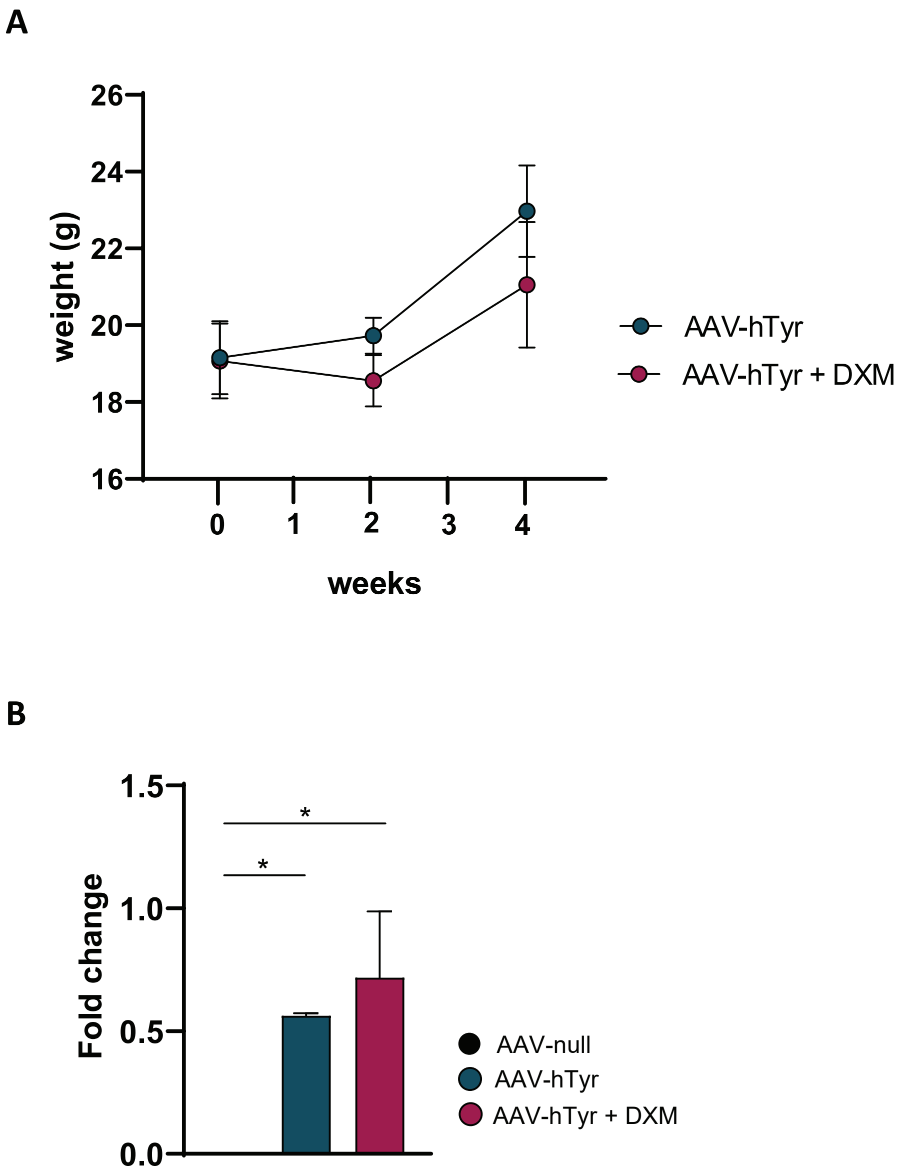

Suppl Figure 2. (A) C57BL6/J mice injected with AAV9-hTyr and treated with dexamethasone (DXM) showed weight loss throughout the 4 weeks of treatment compared to AAV9-hTyr and AAV-null injected mice (n=9 per group). (B) qPCR analysis of gene expression of hTyr in substantia nigra of AAV9-null, AAV9-hTyr and dexamethasone treated AAV9-hTyr C57BL6/J mice (n=3 per group).

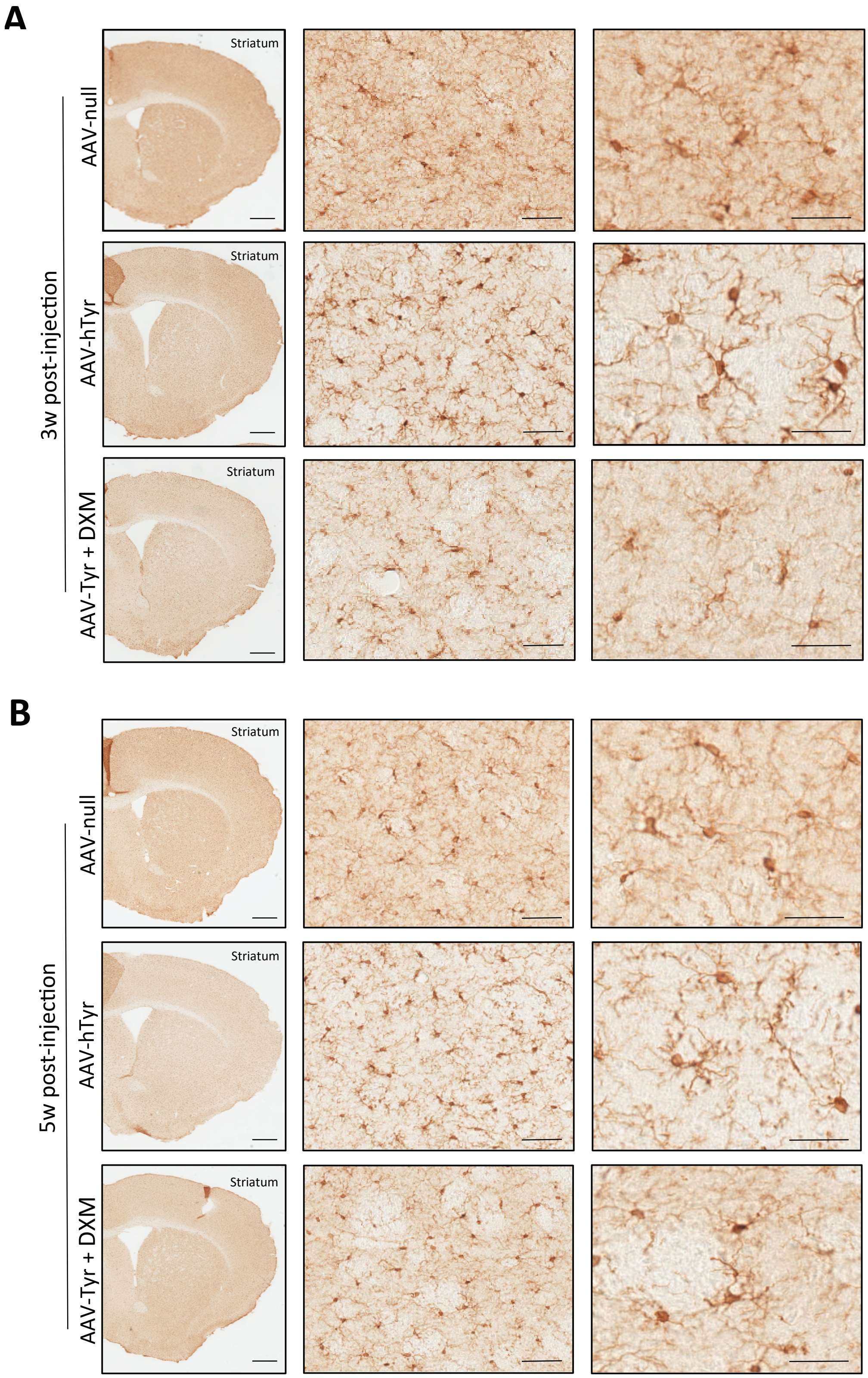

Supp figure 3. There is not microglia activation in the striatum of AAV9-hTyr C57BL6/J mice. Representative images of Iba1 immunostaining in the striatum of C57BL6/J mice (A) 3 and (B) 5 weeks after the injection of AAV9-null and AAV9-hTyr. Magnification bar 1 mm and 200 μm in sets with low magnification and 40 μm in sets with high magnification.

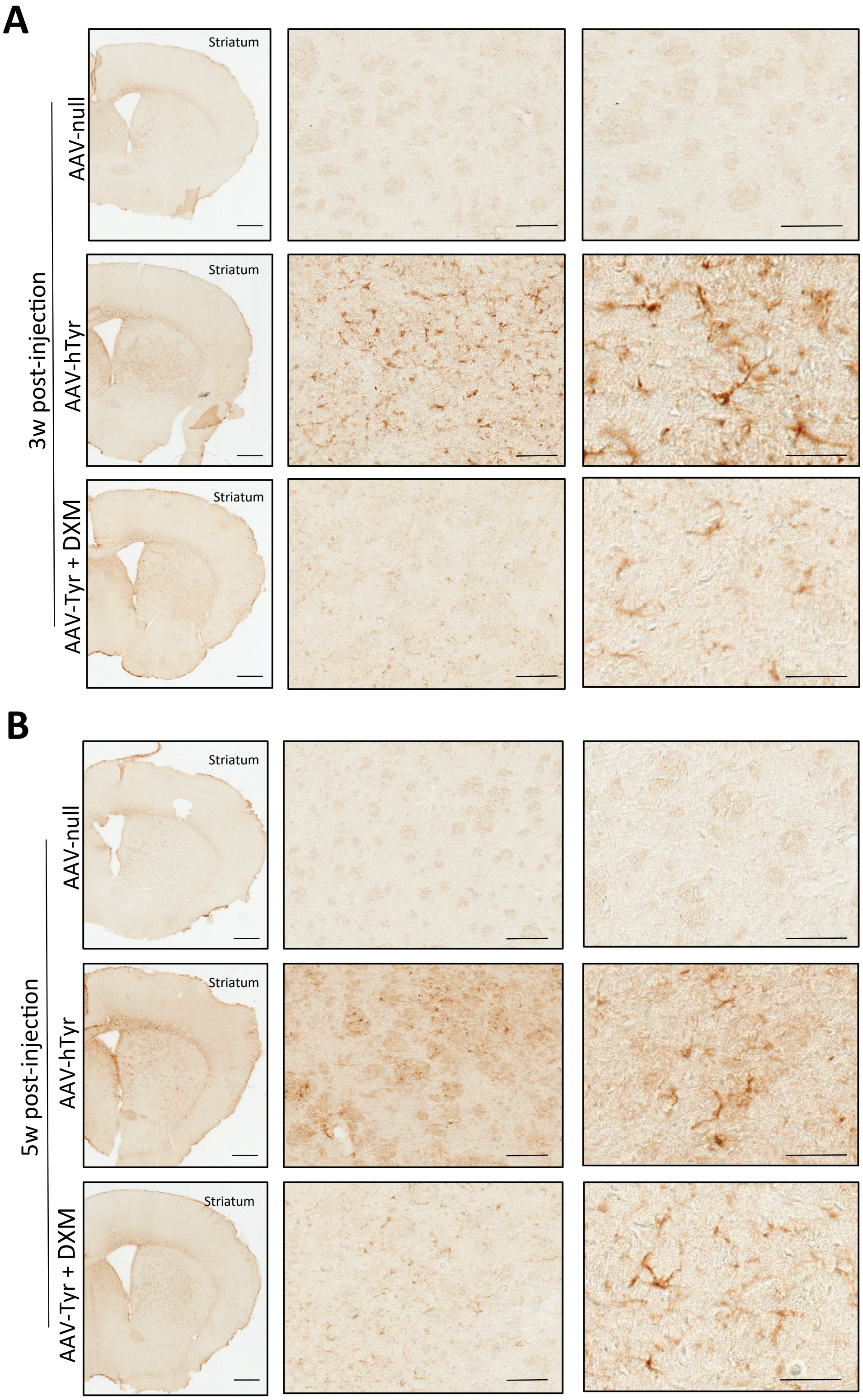

Supp figure 4. Dexamethasone inhibits AAV9-hTyr-induced GFAP activation in the striatum. Representative images of GFAP immunostaining in the striatum of C57BL6/J mice (A) 3 and (B) 5 weeks after the injection of AAV9-null and AAV9-hTyr. Magnification bar 1 mm and 200 µm in sets with low magnification and 40 µm in sets with high magnification.

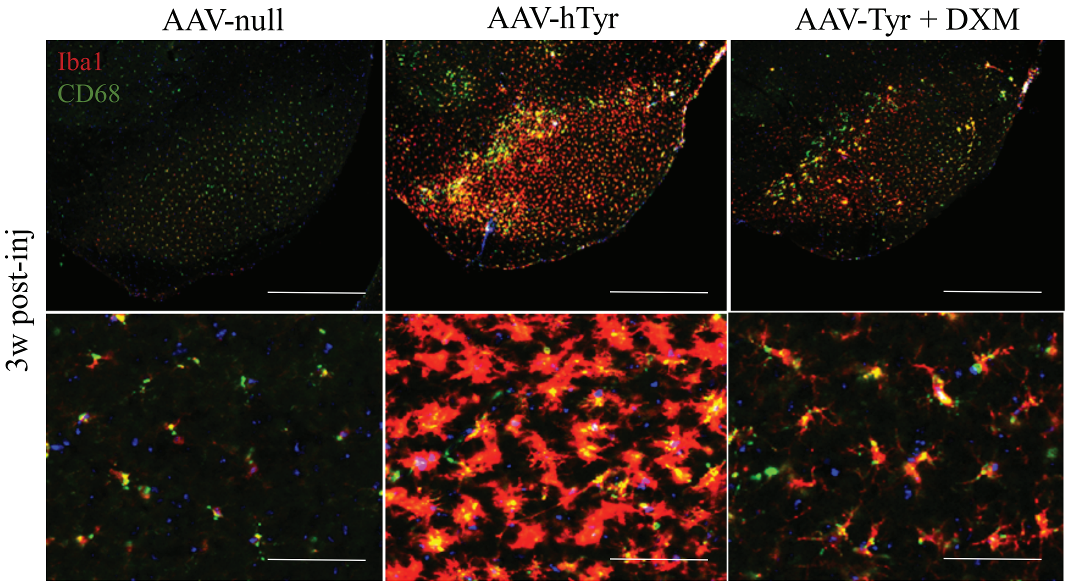

Suppl Figure 5. Iba1 (red) and CD68 (green) double immunofluorescence 3 weeks after AAV9-null and AAV9-hTyr injection treated with vehicle or dexamethasone (DXM). Magnification bar (a) 1mm, and 200 µm in sets with high magnification.
